# Terahertz Metamaterials Inspired by Quantum Phenomena

**DOI:** 10.34133/research.0597

**Published:** 2025-02-03

**Authors:** Ziheng Ren, Yuze Hu, Weibao He, Siyang Hu, Shun Wan, Zhongyi Yu, Wei Liu, Quanlong Yang, Yuri S. Kivshar, Tian Jiang

**Affiliations:** ^1^ College of Advanced Interdisciplinary Studies, National University of Defense Technology, Changsha, China.; ^2^ Institute for Quantum Science and Technology, College of Science, National University of Defense Technology, Changsha, China.; ^3^ School of Physics, Central South University, Changsha, China.; ^4^ Nonlinear Physics Center, Research School of Physics, Australian National University, Canberra, ACT 2615, Australia.

## Abstract

The study of many phenomena in the terahertz (THz) frequency spectral range has emerged as a promising playground in modern science and technology, with extensive applications in high-speed communication, imaging, sensing, and biosensing. Many THz metamaterial designs explore quantum physics phenomena embedded into a classical framework and exhibiting various unexpected behaviors. For spatial THz waves, the effects inspired by quantum phenomena include electromagnetically induced transparency (EIT), Fano resonance, bound states in the continuum (BICs), and exceptional points (EPs) in non-Hermitian systems. They facilitate the realization of extensive functional metadevices and applications. For on-chip THz waves, quantum physics-inspired topological metamaterials, as photonic analogs of topological insulators, can ensure robust, low-loss propagation with suppressed backscattering. These trends open new pathways for high-speed on-chip data transmission and THz photonic integrated circuits, being crucial for the upcoming 6G and 7G wireless communication technologies. Here, we summarize the underlying principles of quantum physics-inspired metamaterials and highlight the latest advances in their application in the THz frequency band, encompassing both spatial and on-chip metadevice realizations.

## Introduction

The terahertz (THz) frequency spectrum, spanning from 0.1 to 10 THz, lies between the microwave and infrared regions of the electromagnetic spectrum. Over the past few decades, THz waves have gained increasing attention due to their distinctive characteristics and enormous potential across various fields, including high-speed communication [1,2], security screening [3], nondestructive testing [4], and biomedical science [5,6], especially pioneering ion channel control and innovative neuron function modulation as novel therapeutic methods for brain diseases [7], and simultaneously achieving quantitative refractive sensing and qualitative fingerprint recognition through an original metasurface-excited surface wave [8]. In addition, THz imaging and spectroscopy serve as effective tools for cancer cell diagnosis, offering the advantage of causing no ionizing harm to biological tissues [9,10]. In particular, the abundant untapped THz spectrum holds tremendous opportunities to revolutionize data transmission, opening the doors for future 6G and beyond wireless communication systems capable of reaching terabits-per-second data rates [11,12].

With these promising prospects, full exploitation of THz wave potential encounters numerous technological obstacles, particularly concerning fundamental THz components, including high-power, miniaturized sources [13], highly efficient modulators, and low-loss on-chip optical interconnect devices. Metamaterials have emerged as an attractive solution due to their capability to flexibly manipulate electromagnetic waves, alleviating existing constraints encountered by THz devices [14]. Quantum physics-inspired metamaterials, which mimic quantum phenomena within a classical framework, unlocking novel functionalities that were previously impossible, have recently attracted considerable interest in photonics. Extensive investigations have been conducted on their potential for manipulating spatial and on-chip THz waves [15–17].

Quantum physics-inspired metamaterials for the manipulation of spatial THz waves exhibit characteristics like electromagnetically induced transparency (EIT) [18,19], Fano resonances [20], bound states in the continuum (BICs) [21], and non-Hermitian exceptional points (EPs) [22,23]. They offer new avenues for the efficient control and confinement of spatial THz waves. The distinctive slow-light phenomenon of the EIT effect enables the reduction of group velocities. Furthermore, the outstanding light confinement and strong light–matter interactions provided by Fano resonances and BICs can promote high-sensitivity sensing, low-threshold lasing, and diverse nonlinear effects. Non-Hermitian EPs, characterized by the coalescence of eigenvalues and eigenstates, can cause unusual phenomena like asymmetric chiral transmission [24], topological phases [25], and square-root topology [26].

In on-chip THz applications, quantum-inspired topological metamaterials, hereafter referred to as "topological metamaterials," have attracted increasing interest. They are the classical electromagnetic counterparts of electronic topological insulators (TIs) and showcase counterintuitive features inaccessible in conventional metamaterials, such as reflection-free unidirectional propagation and robustness against defects [27]. Topological metamaterials can be classified into 3 primary types: quantum Hall (QH) [28], quantum spin Hall (QSH) [29], and quantum valley Hall (QVH) [30] metamaterials. Traditional THz on-chip waveguides frequently suffer severe backscattering loss from sharp bends or structural defects [31], and their limited operational bandwidth constrains their application performance. Topological metamaterials with unique topological propagation mechanisms can surmount these long-standing bottlenecks. This is particularly important for THz photonic integrated circuits and high-speed optical interconnects [32]. Many key THz on-chip components, including but not limited to waveguides, routers, power splitters, antennas, quantum cascade lasers (QCLs), delay lines, and reconfigurable modulators, have been realized by topological metamaterials fabricated on low-loss silicon photonics platforms.

This review contains an overview of recent advances in THz quantum-inspired metamaterials. The first section examines quantum physics-inspired metamaterials for spatial THz wave manipulations, detailing their fundamental physical mechanisms and distinctive characteristics, including EIT, Fano, BIC, and EP types. The second section discusses the applications of quantum physics-inspired metamaterials for modulators, sensors, and various functional devices of spatial THz waves. The third section delves into the fundamental physics underlying the 3 types of QH, QSH, and QVH topological metamaterials, offering illustrative examples to clarify their topological features. Finally, we highlight the current developments in THz on-chip topological metamaterials in communications, routers, power splitters, antennas, QCLs, delay lines, and reconfigurable components.

## Quantum-Inspired Metamaterials for Spatial THz Wave Manipulations

### EIT-type metamaterials

The EIT effect is a quantum optical phenomenon in which an opaque medium becomes transparent at a specific frequency due to destructive interference between quantum states [33]. The vital feature of the EIT effect is the dramatic slowdown of group velocity. EIT metamaterials are typically realized by coupling a bright mode with a dark mode [34–36]. The bright mode refers to the radiative mode, which couples to incident electromagnetic waves and is characterized by a large scattering cross-section. The dark mode is nonradiative and only activates solely through coupling with the bright mode [37]. Similar to EIT in 3-level atomic systems, EIT-like metamaterials also possess 3 states: a ground state ∣0⟩ and 2 excited states ∣1⟩ and ∣2⟩. The transitions ∣0⟩→∣1⟩ and ∣0⟩→∣2⟩ correspond to the "bright" and "dark" mode resonances, respectively. The transition ∣1⟩→∣2⟩ is dependent on the coupling effect. Consequently, 2 transition pathways ∣0⟩→∣1⟩ and ∣0⟩→∣1⟩→∣2⟩→∣1⟩ interact destructively, leading to a sharp transmission peak in a broad absorption background, with a remarkable reduction in group velocity at the transparency window [38,39]. A recent study has shown that a peak group delay of up to 117 ps can be implemented in THz EIT metasurface [40].

### Fano-type metamaterials

Fano resonance, characterized by an asymmetric spectral line shape, arises from the interference between a discrete resonant state and a continuum of states. First theoretically explained by Ugo Fano in 1961, this phenomenon is now widely observed in various photonic systems [41]. Almost any asymmetric resonant state with a quasi-discrete nature and complex frequency can be understood within the framework of Fano resonance.

Fano resonance in metamaterials was initially observed in planar structures composed of asymmetrically split rings [42]. The asymmetry results in a high-quality (Q) leaky mode that couples weakly with free space, with the resonance linewidth determined by the degree of asymmetry [43]. This is closely linked to BIC, which will be discussed in the next section. The high-Q resonances and strong light confinement of Fano metamaterials are crucial for THz sensors [44,45], active switches, and narrowband filters.

### BIC-type metamaterials

In photonic systems, modes oscillating at frequencies outside the continuous spectrum of propagating waves can become trapped as bound states due to the absence of radiative channels. However, when the frequencies fall within the continuous spectrum, they couple with propagating waves and radiate out, becoming "resonances" with finite Q factors. Interestingly, BICs go beyond this conventional wisdom by remaining perfectly localized without any radiation, despite that their frequencies reside within the continuum spectrum [46]. Consequently, BICs are considered trapped modes characterized by suppressed radiation and infinite Q factors​ [21,47–50].​​

The rapid development of metamaterials has catalyzed extensive research on BICs within photonic systems​ [51]. BICs can be classified into 2 primary types based on their formation mechanisms: symmetry-protected BICs [52–55] and interference-based BICs [56]. Symmetry-protected BICs emerge in structures with rotational and/or mirror symmetry, where modes from distinct symmetry classes do not couple with one another [57]. Interference-based BICs arise from destructive interference between radiative modes, as exemplified in Friedrich–Wintgen BICs [58–61]​​.

**Fig. 1.**
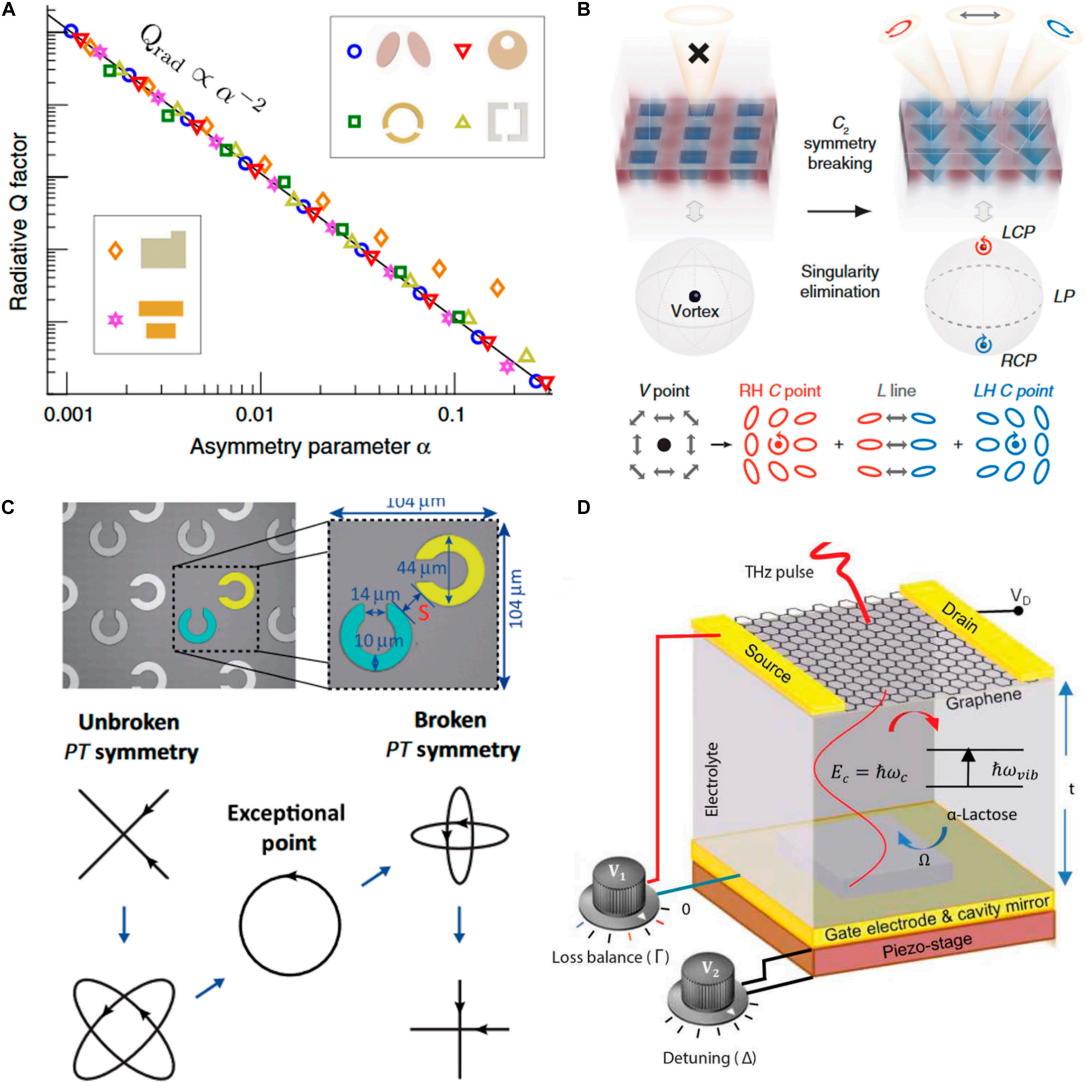
BIC- and EP-type metamaterials. (A) Inverse quadratic dependence of the Q factor of symmetry-broken BIC on the asymmetry parameter *α* [63]. (B) A BIC splits into 2 C points with opposite chirality through breaking in-plane *C*_2_ symmetry [66]. (C) Chiral EP metasurface composed of less and more lossy SRR (top). Evolution of eigenpolarization states from PT-symmetric phase to PT-broken phase; at chiral EP, 2 eigenpolarization states coalesce into one LCP state (bottom) [77]. (D) Electrically tunable loss-induced resonant EP metasurface [81].

In practice, ideal BICs are unattainable due to fabrication imperfections and finite sample sizes. Instead, BICs turn into quasi-BICs, which manifest as high-Q sharp Fano resonances in the scattering​ [62]. Intentional symmetry breaking can transform BICs into quasi-BICs, wherein their Q factors are inversely proportional to the strength of the symmetry breaking perturbation (Fig. 1A) [63]. Furthermore, the Q factors of BICs can be finely tuned by changing the incident wavevector [56].

A compelling aspect of BICs is their topological nature [64]. BICs act as topologically protected singularities, where the surrounding guided modes display far-field polarization vortices that define the topological charges [65]. Breaking in-plane *C*_2_ symmetry can split a BIC into 2 circularly polarized (CP) states (C points) with opposite chirality and identical half-integer topological charges (Fig. 1B) [66]. Further, breaking out-of-plane mirror symmetry can shift C points toward the Γ point, realizing intrinsic chiroptical responses [67–70]. The topological nature of BICs opens new opportunities for polarization control [71], vortex beam generation [72,73], and optical chirality​​ [74].

### EP-type metamaterials

Non-Hermitian systems are open systems that exchange energy with their external environment and are described by non-Hermitian Hamiltonians. Unlike Hermitian systems, which exhibit real-valued eigenvalues, non-Hermitian systems exhibit complex eigenvalues and nonorthogonal eigenstates. The peculiar feature of non-Hermitian systems is the existence of EPs, where multiple eigenvalues and eigenstates coalesce, causing the breakdown in the linear independence of eigenstates and the reduction in the dimensionality of the eigenspace. This contrasts with diabolic points (DPs) of Hermitian systems, where only eigenvalues are degenerate, but eigenstates remain orthogonal. The formation of EPs can be explained by coupled mode theory, as in [75]. In non-Hermitian systems that obey parity-time (PT) symmetry or anti-PT symmetry, EPs emerge at the transition between the symmetric and the broken phases. Recent reviews offer detailed insights into EPs in non-Hermitian photonic systems with PT or anti-PT symmetry [22,76].

Metamaterials have been proven to serve as a fertile ground for constructing controllable non-Hermitian systems and EPs. For instance, EP has been demonstrated in a metasurface composed of 2 coupled split-ring resonators (SRRs) with different nonradiative loss rates (Fig. 1C) [77]. As the mode coupling increases, the system switches from a PT-symmetric phase to a PT-broken phase, which is associated with the evolution of the eigenpolarization states. At the EP, the 2 eigenpolarization states coalesce into a left-circularly polarized (LCP) state, marking a highly asymmetric transmission of CP light, i.e., *T_ll_* = *T_rr_*, and *T_rl_* is marked suppressed compared to *T_lr_* [where *T*_*ll* (*rr*)_ denotes the transmission from LCP (RCP) to LCP (RCP) and *T*_*rl* (*lr*)_ denotes the transmitted CP conversion from LCP (RCP) to RCP (LCP), respectively]. This type of EPs, constructed using a non-Hermitian Jones matrix, is referred to as chiral EPs [78], with their eigenstates positioned at the poles of the Poincaré sphere. An intriguing phenomenon occurs when encircling the EP in the parameter space: One circular polarization conversion channel accumulates a 2π phase, while the other remains zero, revealing an exceptional topological phase​ [79–81].

Remarkably, achieving EPs does not require strict PT symmetry with balanced gain and loss. EPs can be realized in equivalent PT-symmetric systems embedded in a lossy background, which does not impact the phase transitions and eigenstates of the PT-symmetric system. Thus, it is possible to observe loss-induced phase transition and EP in a lossy system without the need for multiple structural samples [24,80–85]. For instance, a dual-voltage tunable THz metasurface has been proposed to achieve EP in light–matter interactions (Fig. 1D) [81]. This metasurface integrates lactose crystals, known for their collective intermolecular vibrations in the THz band, with a graphene-based tunable resonator. The loss imbalance and frequency detuning between the vibration and cavity-like modes can be tuned by voltages *V*_1_ and *V*_2_. In the parameter space defined by *V*_1_ and *V*_2_, the 2 complex eigenfrequencies coalesce, resulting in a resonant EP at the intersection of 2 Riemann sheets. This resonant EP is observable in the reflection spectra at the transition point where 2 split resonances merge into one [86,87].

The exotic phenomena associated with EPs in metamaterials present exciting opportunities for THz metadevices, with potential applications in polarization modulation, sensing, and wavefront control.

## Applications of Quantum-Inspired Metamaterials for Spatial THz Waves

### Modulators

The weak electromagnetic response of conventional materials in the THz frequency range highlights the need for efficient THz modulators to advance practical applications. Metamaterials with tunable electromagnetic properties provide an effective solution for manipulating various THz wave parameters, including amplitude, phase, and polarization. THz modulators are generally categorized into passive and active types. Passive modulators have fixed responses and limited flexibility. In contrast, active reconfigurable modulators, which integrate metamaterials with active materials [such as two-dimensional (2D) materials [88,89], superconductors [90], liquid crystals [91], phase-change materials [92,93], TIs [94], and perovskites [95]] are capable of dynamically responding to external electric, optical, and thermal stimuli. THz modulators are rapidly evolving, and several studies have reviewed these metadevices based on modulation methods and material types [17,96–99]. Here, we focus on the novel approaches and potentials of quantum-inspired metamaterials in THz spatial light modulation technologies.

EIT metamaterials offer notable advantages in THz modulation owing to their slow-light effects and enhanced light–matter interactions. For instance, integrating photoactive silicon islands into the EIT metasurface allows active tuning of THz wave group velocity and amplitude via optical pump excitation [38]. This is driven by changes in the dark mode damping rates from increased photoconductivity. Similarly, loss-induced modulation with other active materials follows this principle [39,94,100–109].

**Fig. 2.**
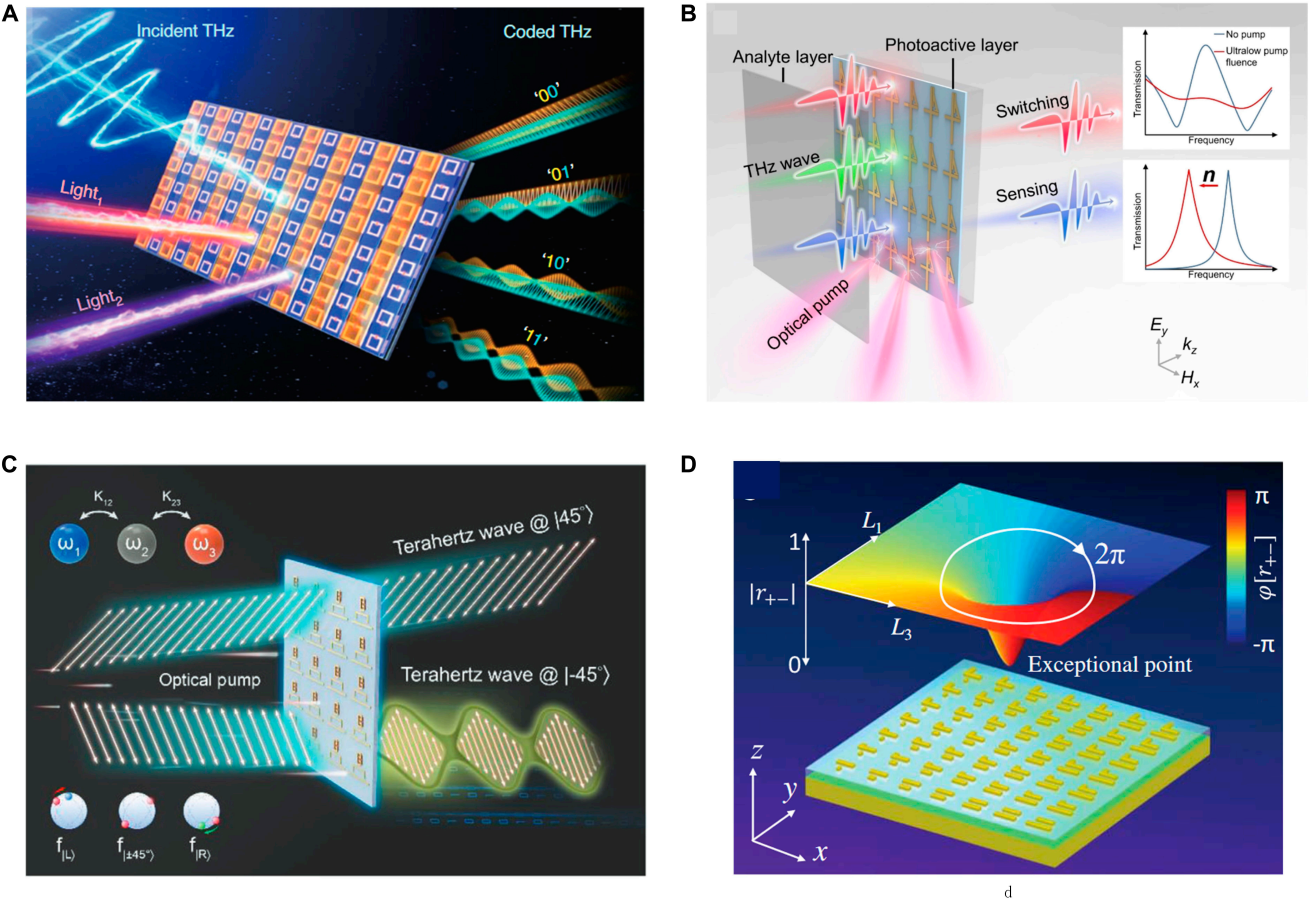
THz quantum-inspired metamaterial modulators. (A) EIT metasurface for 2-bit dual-channel THz encoding, where the 4 encoding states are controlled by pump laser wavelengths [110]. (B) Ultra-low pump threshold THz switch induced by strong coupling between quasi-BIC and dipole mode [117]. (C) Asymmetric linearly polarized THz wave modulation enabled by the emergence of a pair of anti-chiral EPs [84]. (D) Topological 2π phase of CP conversion channel around the EP in 2D parameter space [25].

The modes available in EIT metamaterials offer additional freedom for information encoding. In [110], an optically programmable THz modulator has been demonstrated by integrating dual-EIT metasurface with a periodically arranged distributed Bragg reflector (Fig. 2A). By selectively using pump colors to suppress the dark mode resonance, it can independently modulate 2 ultrafast channels, enabling rapid switching between 4 distinct encoding states. Additionally, the EIT coupling effect can be used to modulate nonlinear THz generation [111]. Nonchiral coupled meta-molecules generate THz waves with linearly polarized pumping, while chiral meta-molecules react to the chirality of pump light. Chiral meta-molecules arranged in clockwise or counterclockwise orientations produce broadband THz beams with distinct orbital angular momentum. Microelectromechanical system (MEMS) integrated EIT metamaterial is a promising way to miniaturize and integrate into real optical systems through direct electrical control [112].

Research on optimizing THz modulators using Fano resonances and BICs has rapidly increased [113,114]. BICs can effectively lower the pump threshold for THz switches. One approach employs all-dielectric THz metasurfaces that support high-Q supercavity BICs, enabling THz switching with very low pump fluence [115,116]. A plasmonic metasurface, where a low-radiation-loss quasi-BIC strongly couples with a high-radiation-loss dipole mode, has proven a reduced pump threshold by an order of magnitude [117] (Fig. 2B). These BIC-enabled low-power switches are promising components for next-generation THz free-space communication systems.

The radiation channels of BICs can also be dynamically activated or deactivated [118–121]. For instance, vanadium dioxide (VO_2_) embedded in SRRs can break the in-plane *C*_2_ symmetry and consequently trigger the leakage of a hidden BIC in the EIT window [122]. This provides a new method to tailor THz spectral line shapes and tune individual resonances actively. The topological features of BICs facilitate efficient modulations of THz wave polarization states and spin-orbit coupling. Experimental efforts have demonstrated complete polarization-coherent control by leveraging the topological polarization features and angle-dependent properties of symmetry-protected BICs in silicon photonic crystal slabs (PCSs) [15]. The output polarization state can be tuned across the entire Poincaré sphere by adjusting the incidence angle and the in-plane rotational angle of the sample.

In PT-symmetric non-Hermitian systems, abrupt changes in eigenstates occur when going through an EP. For chiral EPs, the coalescence of eigenpolarization states reduces the dimensionality of the eigenspace, resulting in exotic transmission behaviors. This phenomenon offers a novel approach to manipulating the THz wave. For instance, a THz linear-to-circular polarization modulation has been constructed in a non-Hermitian metasurface [85], leveraging the asymmetry in circular polarization conversion near EP​. Similarly, asymmetric transmission can be accessed by a chiral EP metasurface [80]. When incident from the frontward, *T*_*lr*​_ is zero, while from the backward, *T*_*rl*​_ is nonzero​. It has also been shown that the loss-induced merging of a pair of independent anti-chiral EPs can lead to asymmetric modulation in 2 orthogonal linearly polarized waves [84]. Under optical pumping, the Ge-hybrid non-Hermitian metasurface can fully modulate one linear polarization wave while leaving the orthogonal linear polarization unaffected​ (Fig. 2C)​.

At the chiral EP, one circular polarization conversion experiences annihilation, resulting in a zero point. Encircling this zero point in a 2D parameter space can generate a 2π phase shift (Fig. 2D), topologically protected by the singularity and independent of the encircling path. In contrast, the other circular polarization conversion does not exhibit a zero point, and its phase remains unchanged. This differs fundamentally from the traditional Pancharatnam–Berry (PB) phase. By combining the topological phase around the EP and the PB phase, independent control of the wavefronts of each CP wave has been achieved [25]. Decoupling the 2 circular polarizations can create distinct holographic projections, where image "A" is produced for LCP light and image "B" is produced for RCP light. Utilizing topological phase control, a wavelength division multiplexing device has been developed, which projects separate holographic images on the same channel at 2 different wavelengths [123]. Notably, the asymmetric behavior of EPs can be generalized to operate under arbitrary polarization states, not limited to a single circular polarization. Effective vectorial wavefront shaping and asymmetric meta-holograms across full-polarization states can be achieved by constructing a pair of EPs with opposite handedness in the metasurface [124].

Table 1 summarizes recent works in quantum-inspired THz metamaterial modulators, highlighting their various performance metrics, e.g., modulation depth, modulation speed, and group delay.

**Table 1.** Performance of various quantum-inspired THz modulators

Quantum phenomena	Year	Active material	Modulation depth	Modulation speed	Group delay
EIT [39]	2018	Graphene	25%	/	3.3 ps
EIT [104]	2022	Germanium	90%	10 ps	/
EIT [283]	2023	MoTe_2_	77%	<300 ps (half-recovery state)	4.6 ps
EIT [110]	2024	Silicon	/	1 ns	/
EIT [284]	2024	Organic mixed ion-electron conductors	65%	/	/
EIT [108]	2020	Transition metal dichalcogenides WSe_2_	43%	8 ps	6 ps
EIT [94]	2021	Topological insulators Bi_2_Se_3_	31%	9.5 ps	2.7 ps
BIC/EIT [122]	2022	VO_2_, germanium	100% (normalized)	7 ps	1.5 ps
BIC/Fano [113]	2020	Perovskites	93% (normalized)	/	/
BIC/Fano [95]	2019	Solution-processed lead iodide PbI_2_	100% (normalized)	<75 ps	/
BIC/Fano [285]	2019	Silicon	/	2.6 ns	/
BIC/Fano [118]	2021	Germanium	75%	7 ps	/
BIC/Fano [286]	2022	Germanium	56.3%	10 ps	/
BIC/Fano [117]	2024	Germanium	100% (normalized)	7 ps	/
EP [84]	2024	Germanium	70%	9 ps	/

### Sensors

THz waves possess unique properties such as low energy, molecular fingerprinting, and high penetration, making them ideal for biochemical sensing and substance recognition. However, their relatively long wavelength limits interaction with trace biomolecules, reducing sensing sensitivity. Sensing sensitivity quantifies a sensor's ability to detect small changes in target substances—such as molecular concentration—reflected in measurable variations in the spectrum, absorption, or phase. EIT, Fano, and BIC-type metamaterials can spatially and temporally confine electromagnetic excitation energy within subwavelength volumes [125,126], enhancing both the duration and intensity of interactions between THz waves and analytes and amplifying the spectral information embedded in the substance.

Most biological components exist in aqueous environments, making liquid-based biosensing highly critical. The refractive index and the absorption coefficient of biochemical solutions change with solute concentration. By overlaying a metasurface with these solutions or integrating them into microfluidic channels, concentration levels can be accurately measured through spectroscopic detection. Compared to direct drop-casting methods, microfluidic sensors with fixed chambers ensure a consistent volume of analyte, as the solution thickness can affect sensing results. They prevent variations in surface solution thickness caused by evaporation. Moreover, the ultrathin microfluidic sensing layer (on the order of tens of micrometers) reduces the attenuation of THz waves by water, making the substance information to be more reflected in detection signals. It is important to consider that the materials used for microfluidic channels should be transparent to THz waves or exhibit minimal absorption, such as quartz [127] or polytetrafluoroethylene [128]. Additionally, the microfluidic chambers need to be fully sealed to prevent liquid leakage and evaporation.

**Fig. 3.**
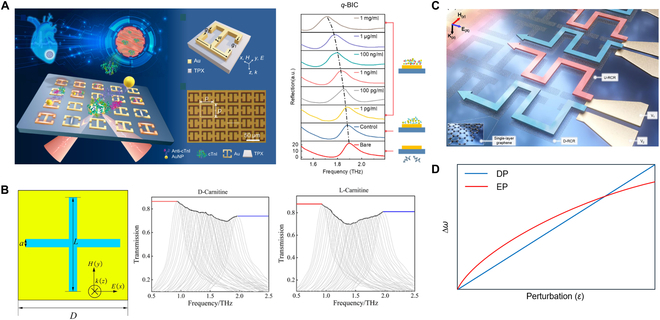
THz quantum-inspired metamaterial sensors. (A) BIC-mediated THz metasensor for antibody concentration detection [129]. (B) Broadband frequency multiplexing metasurfaces for trace chiral enantiomer detection [146]. (C) Graphene-integrated single-pixel reconfigurable EIT metasurface for ultra-wideband fingerprint enhancement sensing of trace analytes [150]. (D) Resonant EP metasensor exhibits frequency splitting proportional to the square root of the perturbation $\;\Delta \omega_{\mathrm{EP}}\propto \sqrt{\varepsilon }$.

A BIC-mediated THz biosensor has been proposed to detect conjugated antibody at concentrations as low as 0.5 pg/ml [129] (Fig. 3A). The quasi-BIC's frequency redshifts progressively with increasing concentration. EIT and Fano/BIC metamaterials have been used for the concentration detection of amino acids [130–132], proteins [133], pesticides [134], cancer cells [135], DNA [127], and various chemical molecules [136–138]. The accuracy of optical sensors is often influenced by environmental noise. Novel methods by engineering 2 ps-delayed transmission spectra from a single laser-controlled metasurface are innovatively proposed and demonstrated to achieve original calibration-free, high-precision, and robust sensing [139]. However, refractive index-based sensing is limited to quantifying pre-identified substances and lacks specific molecular recognition capabilities.

Most polar molecules and biomacromolecules exhibit vibrational or rotational spectra in the THz range, providing unique fingerprint spectra for molecular identification. When interacting with target molecules, sharp resonances deform due to molecular absorption [140,141]. However, this method requires the resonance frequency to match the target molecules, reducing its applicability for various molecular identification. Multiplexed devices have been designed to generate broad, comb-like spectral resonances to address this limitation. These devices can map fingerprint features at higher amplitudes than traditional absorption spectroscopy. One approach involves continuously scaling dielectric unit cells to tune quasi-BIC frequencies, yielding discrete high-Q resonances with high spectral resolution and near-field enhancement [142]. Metasurfaces with discrete resonance frequencies are pixelated in a multi-pixel array. By comparing spectroscopic imaging readouts before and after coating the analyte, the molecular fingerprint spectra are extracted. Another method to achieve high-Q resonances over a broad spectral range includes adjusting the incident angle and polarization [143]; total signal strength at each incident angle correlates with molecular absorption intensity.

Enhanced THz trace fingerprint detection can be achieved using multiplexing strategies [144–149]. For example, a frequency-selective metasensor has been engineered to enhance and retrieve the fingerprints of chiral enantiomers d-carnitine and l-carnitine [146]. Comb-like resonances in the range of 0.95 to 2 THz can be generated by tuning the cross slots (Fig. 3B). Although the Q factors of these resonances are relatively low, substantial field enhancement can boost absorption-induced transparency. A set of 140 evenly spaced sharp quasi-BIC resonances within the 500- to 750-GHz range have been demonstrated by scaling the pixelated THz metagrating array [149]. The compressed resonance can further improve fingerprint spectra resolution and offer the potential to distinguish substances with similar THz fingerprints. Moreover, a dielectric metagrating with angle-multiplexed spectra has been developed to enhance detection sensitivity for trace amounts of α-lactose and tantalum oxide fingerprints, achieving a maximum enhancement factor of up to 98 times compared to conventional methods [148].

In addition to broadband trace detection using multi-pixel or angle multiplexing, another method incorporates tunable graphene into the resonator, modulating the resonance frequency linearly with the Fermi level of graphene [147]. A single-pixel reconfigurable graphene EIT metasurface with a dual-tuning scheme for controlling the Fermi level is proposed (Fig. 3C) [150]. Under synchronized voltage tuning, the metasurface enables ultra-broadband (0.5 to 2.0 THz) enhanced THz fingerprint detection of multiple trace molecules, as well as precise identification of chiral pharmaceutical enantiomers in a single-pixel retrieval scheme. The active tuning scheme allows a single metasurface pixel to be adjusted across multiple characteristic absorption spectra, promoting device miniaturization.

Systems operating at or near EPs exhibit unique behaviors. For an *n*th-order EP (*n* eigenvalues and eigenstates degenerate), the splitting of the eigenvalues is proportional to an *n*th root of the perturbation strength *ε* [151]. This principle was initially proposed for nanoparticle sensing with a microring resonator coupled to a waveguide [152–154]. A resonant EP metasensor has also demonstrated this principle [87]. When the metasensor operates around an EP, the frequency splitting of the 2 coalesced modes is proportional to the square root of perturbation $\Delta \omega_{\mathrm{EP}}\propto \sqrt{\varepsilon }$ (Fig. 3D). The DP sensor shows frequency splitting depends linearly on the perturbation $\Delta \omega_{\mathrm{DP}}\propto \sqrt{\varepsilon }$. EP sensors exhibit larger splitting with small perturbations, while DP sensors demonstrate greater frequency splitting with larger perturbations. Recent theoretical studies indicate that multiple BICs can merge into a single EP, forming a novel EP-BIC [155]. This state inherits both the infinite radiative Q factors of BICs and the enhanced sensitivity at the EPs.

Table 2 presents a summary of quantum-inspired THz metasensors, focusing on their sensitivity, detection limits, and enhancement factors.

**Table 2.** Performance of various quantum-inspired THz sensors

Quantum phenomena	Year	Experimental analyte	Sensitivity	Limit of detection	Enhancement factor
EIT [287]	2024	Basal cell carcinoma	550 GHz/RIU	/	/
EIT [135]	2022	Colorectal cells	118.4 GHz/RIU	/	/
EIT [288]	2021	Glioma cell	496.01 GHz/RIU 248.75 kHz/cell/ml	/	/
EIT [150]	2024	γ-Aminobutyric acid	220 GHz/RIU	0.64 μg/mm^2^	54.9 times
BIC/Fano [289]	2023	Bovine serum albumin	12 GHz/mg/ml	0.17 mg/ml	/
BIC/Fano [125]	2021	/	489 GHz/RIU	/	/
BIC/Fano [290]	2021	/	39 GHz/RIU	/	/
BIC/Fano [291]	2023	Trace homocysteine molecules	420 GHz/RIU	12.5 pmol/μl	/
BIC/Fano [129]	2024	Antibody–AuNPs	560 GHz/RIU	0.5 pg/ml	/
BIC/Fano [146]	2023	α-Lactose	/	/	7.3 times
BIC/Fano [148]	2024	α-Lactose/Ta_2_O_5_	/	/	98 times
BIC/Fano [144]	2022	α-Lactose	/	/	330 times
EP [86]	2024	PI films	9,046 GHz/RIU/mm	/	/

### Other devices

**Fig. 4.**
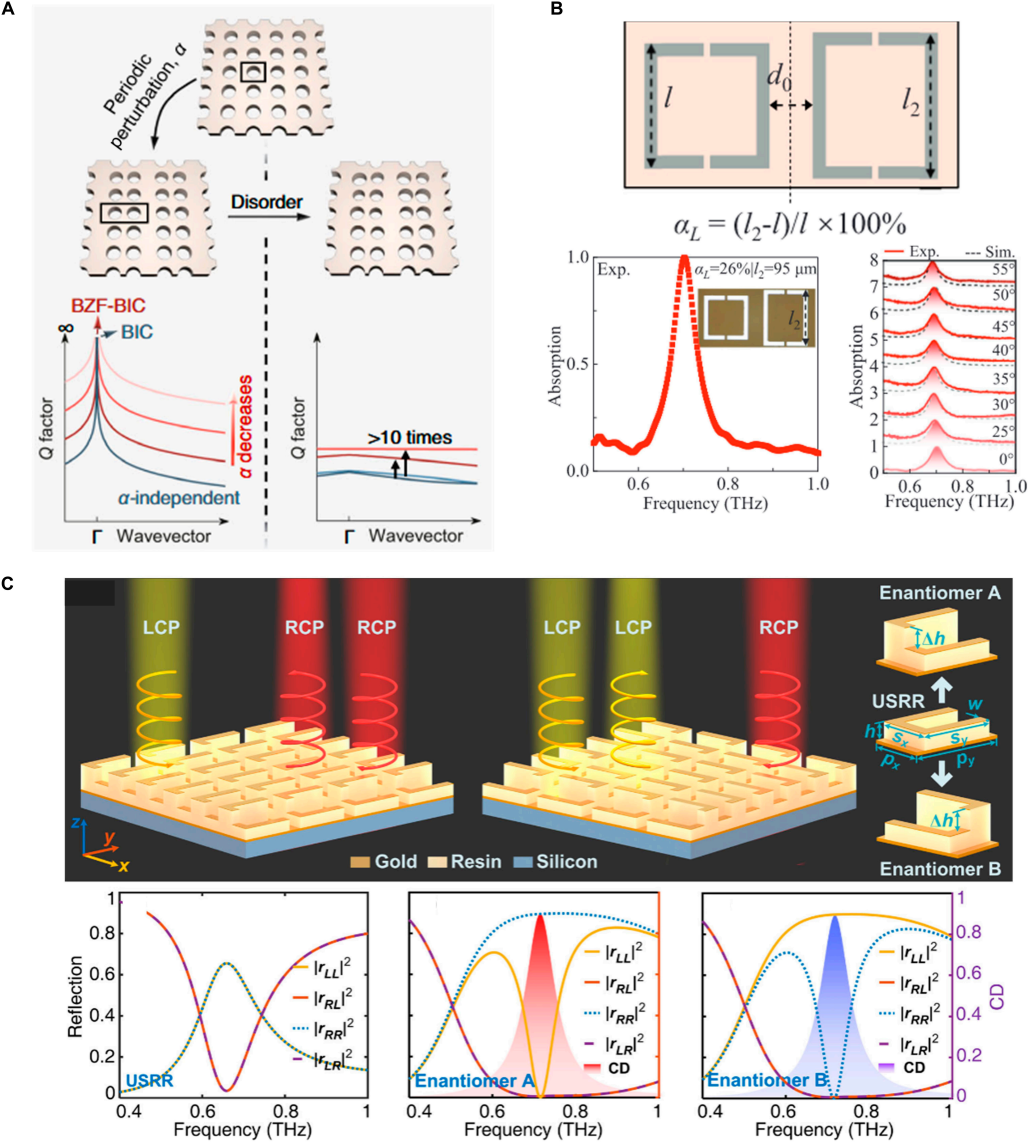
BZF-BICs and chiral THz metamaterials. (A) Implementing BZF-BICs in a PCS with a doubled period size achieved through the introduction of periodic perturbations [158]. (B) Wide-angle flexible perfect absorber empowered by BZF-BICs [159]. (C) Chiral metasurface with broken in-plane and out-of-plane symmetries [162].

Enhanced THz emission in a nonlinear optical film can be realized using a BIC metasurface, which can tightly confine the pump energy within the film [156]. The Q factor of BIC drops sharply as one moves away from the BIC point in momentum space, following a quadratic dependence $Q\propto 1/\left(k-k_{\mathrm{BIC}}\right)$. This indicates that high-Q factors are achievable within a limited region around the BIC point. Brillouin zone folding presents a solution by allowing BICs with robust ultra-high Q factors over an extended range of momentum space [157–160]. This can be realized by introducing periodic perturbations into PCSs Fig. 4A. Consequently, guided modes originally below the light cone are folded into the light cone, leading to Brillouin zone folding-induced BICs (BZF-BICs). BZF-BICs' Q factors depend on both the perturbation factor *α* and the wavevector *k* and follow the relationship $Q_{BZF-\mathrm{BIC}}\propto 1/k^{2}a^{2}$. BZF-BIC has been used to design the THz flexible absorber capable of broadband, near-perfect absorption over a wide field of view of ±55° Fig. 4B [159]. Moreover, the suppressed radiation of BICs can be used to reduce the required pump area for THz quantum cascade microlaser arrays [161]. C points from BIC splitting exhibit a strong chiral response and offer a feasible path for advancing THz chiral photonics. A chiral metasurface can be designed using resonators with broken in-plane and out-of-plane symmetries Fig. 4C [162]. Recently, intrinsic chiral BICs have been demonstrated through engineering in-plane asymmetry perturbations and coupling between transverse electric (TE)- and transverse magnetic (TM)-like BICs [163,164].

A notable trend is the progression of quantum technology via metamaterials. The quantum metamaterials provide an innovative platform for quantum sources [165,166], quantum manipulation [167], and sensing [168]. Metasurfaces can relax phase-matching requirements, in conjunction with high-Q BICs, enabling the enhanced spontaneous emission of entangled photons and photon pairs [169]. Metamaterials have been proposed to facilitate the manipulation of photonic quantum states and the interactions among them [170]. Moreover, they have exceptional proficiency in weak-signal sensing, presenting great potential for integration with quantum detection technologies.

## On-Chip Quantum-Inspired THz Metamaterials: Topological Photonic Insulators

Topology is a mathematical discipline that examines geometric qualities that remain unchanged throughout continuous transformations, including connectedness and the count of holes. The notion of topological metamaterials [171–173], also known as photonic topological insulators (PTIs), originates from condensed matter physics, particularly in the study of TIs [174], which function as insulators in their interior while supporting conductive states along their surfaces [175]. The surface states are protected by topological properties, supporting unidirectional propagation without backscattering, even in the presence of defects [176]. Motivated by this, researchers investigated analogous topology in photonic systems, given that these systems exhibit similar mathematical structures. The following discusses the fundamental physics of PTIs and different band topologies.

### Fundamentals of PTIs

Prior to addressing PTIs, it is imperative to elucidate the notion of topological invariants, which define the global characteristics of eigenfunctions within an energy band. In periodic metamaterials, topological invariants are generally defined by the Chern number. The Chern number of a 2D dispersion band is obtained from the integral of the Berry curvature throughout the entire Brillouin zone, mathematically represented as:

$$C_{n}=\frac{1}{2\pi }\iint_{\mathrm{BZ}}\Omega_{n}\left(k_{x},\;k_{y}\right)d^{2}k$$ (1)

where $\Omega_{n}\left(k_{x},\;k_{y}\right)=\partial A_{y}^{n}/\partial k_{x}-\partial A_{x}^{n}/\partial k_{y}$ presents the Berry curvature, $A_{n}\left(k\right)=i\langle \Psi_{n}\left(k\right)|\nabla_{k}|\Psi_{n}\left(k\right)\rangle$ is the Berry connection, and $\Psi_{n}\left(k\right)$ denotes the eigenfunction of the *n*th band at wavevector *k*. Systems with zero Chern numbers across all bands are regarded as topologically trivial, while those with nonzero Chern numbers are topologically nontrivial.

According to the bulk-boundary correspondence, edge states arise at the boundary where two topologically distinct systems interface. As the topological features of the bulk change gradually, the wavefunction must maintain continuity, forming gapless states that traverse the bulk bands and facilitate a continuous spectral transition. The edge states are guaranteed by the disparity in Chern numbers between the two bulk bands, which also dictates the quantity of edge states. The group velocities of these edge states are uniformly either positive or negative. This permits light to propagate along the boundary unidirectionally. In contrast to photonic crystal line-defect waveguides [31], which depend on bandgap for transmission, topological edge states provide a robust and defect-resistant pathway for steering electromagnetic waves.

Based on the band topology, PTIs can be classified into three categories: the QH phase, which requires broken T symmetry; the QSH phase, facilitated by spin-orbit coupling; and the QVH phase, characterized by broken P symmetry. Each insulating phase originates from different band topologies and exhibits distinctive topological edge states. Besides, Floquet TIs, with temporal modulation as well as broken T symmetry, can also induce one-way edge states [177].

### QH PTIs

When T symmetry is preserved, the Berry curvature satisfies $\Omega_{n}\left(k\right)=-\Omega_{n}\left(-k\right)$, resulting in its integral being zero across the whole Brillouin zone for all bands. However, when T symmetry is disrupted while maintaining P symmetry, the Berry curvature adheres to $\Omega_{n}\left(k\right)=\Omega_{n}\left(-k\right)$, resulting in bands with nonzero Chern numbers and the emergence of the QH phase.

**Fig. 5.**
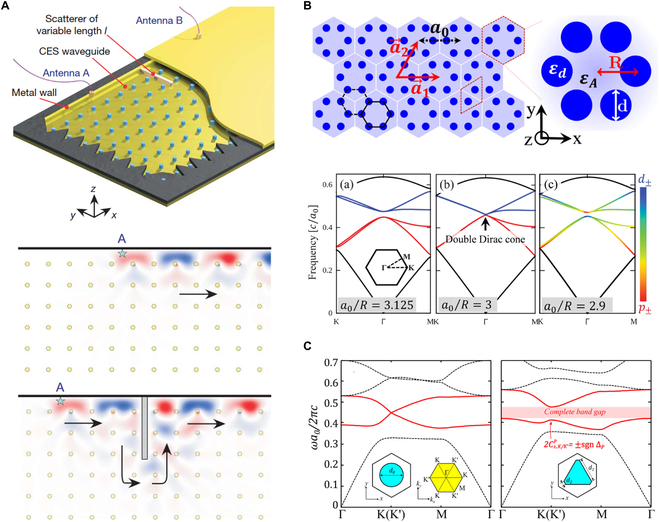
PTIs. (A) QH PTI [28]. Experimental setup for observing the chiral edge state in a QH metamaterial with brokenT symmetry (top). Topologically protected chiral edge states propagate unidirectionally to the right along the metal wall and can navigate around obstacles without backscattering (bottom). (B) QSH PTI [181]. Schematic of the lattice of hexagonal clusters composed of 6 cylinders of dielectric material (top). Frequency–momentum dispersion of TM modes in contracted, honeycomb, and expanded hexagonal clusters (bottom). (C) QVH PTI. Band structure of the *C*_6_ lattice (left). Bulk bands are opened, and the QVH phase appears within theP-broken lattice (right) [185].

The foundational theory for photonic analogs of the QH effect was first proposed by Haldane and Raghu [178]. Subsequently, QH PTI was experimentally demonstrated utilizing magneto-optical photonic crystals (Fig. 5A, top panel) [28]. An external magnetic field is applied to break T symmetry, causing a gap between the second and third TM bands. The two bands possess different nonzero Chern numbers. According to bulk-boundary correspondence, a chiral edge state appears at energies within the bulk bandgap, propagating exclusively to the right along the boundary while exhibiting no leftward propagation and smoothly circumventing barriers (Fig. 5A, bottom panel).

### QSH PTIs

Practically, breaking T symmetry to realize the QH effect is challenging. Conversely, the QSH effect keeps T symmetry, which can be seen as the superposition of two QH effects with opposite magnetic field orientations. It originates from spin-momentum locking, where edge states propagate in opposite directions corresponding to opposite spin orientations [179,180]. The QSH phase is identified by the $Z_{2}$ invariant or spin-Chern number. The Chern numbers of spin-up $C_{↑}$ and spin-down $C_{↓}$ states are equal in magnitude but opposite in sign, summing to zero ($C_{↑}+C_{↓}=0$). Unlike fermions, such as electrons with half-integer spin (1/2), photons are bosons with integer spin (1) and lack intrinsic Kramers degeneracy. Kramers' theorem necessitates $T^{2}=-1$ for the QSH effect, a condition that electrons fulfill. However, photons obey $T^{2}=+1$, hence precluding Kramers degeneracy in photonic systems. However, pseudospin and pseudo-T symmetry can be introduced to generate conjugate pseudospin-up and pseudospin-down states. This leads to a Kramers-like degeneracy under artificial gauge symmetries, thereby realizing the QSH effect in photonic systems.

A straightforward method to achieve the photonic QSH effect has been proposed using dielectric photonic crystals [181]. As shown in Fig. 5B (top panel), six dielectric cylinders surrounded by air are set into a honeycomb lattice. The TM modes of hexagonal artificial atoms are analogous to electronic *p-* and *d*-wave orbitals. The pseudospin states *p*_±_ and *d*_±_ arise from combinations of these orbitals, corresponding to magnetic fields with positive and negative angular momenta, respectively. As the side length of the hexagonal lattice expands, the degenerate Dirac point opens, resulting in a trivial bandgap with *p*_±_ and *d*_±_ states occupying the bands beneath and above the gap, respectively. Conversely, diminishing the side length leads to a band inversion near the Dirac point, putting *p*_±_ states in the upper band and *d*_±_ states in the lower band (Fig. 5B, bottom panel). This inversion produces topologically nontrivial bands characterized by nonzero spin Chern numbers.

### QVH PTIs

Alongside the QH and QSH phases, the subsequent advances have introduced an additional PTI termed the QVH PTI [182]. A valley denotes the extrema of the valence and conduction bands positioned at the high symmetry points of the Brillouin zone. The QVH phase results from the breaking of P symmetry, which lifts the degeneracy of the massless Dirac cones, converting them into massive Dirac cones at the *K* and *K*′ valleys. The Bloch states at the *K* and *K*′ valleys display opposing self-rotation, leading to valley-locked pseudospins with orbital magnetic moments. Although the Berry curvature integrated over the Brillouin zone totals zero, it exhibits nonzero, opposite values in the vicinity of the *K* and *K*′ valleys [183]. The valley Chern number, a topological invariant, is defined as the integral of the Berry curvature in proximity to each valley, taking values of ±1/2 under conditions of small strength of the P symmetry breaking. This leads to valley-distinctive physics, wherein the behavior of photons is contingent upon the valley.

A domain wall can be constructed between two PTIs with opposite valley Chern numbers. The valley Chern number difference across the domain wall at the *K* (*K*′) valley is ±1, giving rise to a pair of valley-polarized topological kink states within the bandgap. The propagation direction of kink states is locked to the valley, with opposite group velocities at the *K* and *K*′ valleys, illustrating valley-selective transport. However, intervalley scattering renders kink states less robust to perturbations than chiral and helical edge states [184]. Notwithstanding this, their principal advantages are in effective transmission through sharp bends, the simplicity of design, and large operating bandwidth [183].

The photonic QVH effect was first proposed in an all-dielectric photonic crystal consisting of round silicon rods organized in a hexagonal lattice [185]. Deforming the rods from circular to triangular cross-sections breaks the P symmetry, thus lifting the Dirac degeneracy and opening a bandgap within the TE bulk bands (Fig. 5C). This yields nonzero valley Chern numbers, leading to a nontrivial valley topological phase. Multiple lattice configurations with broken P symmetry have been employed to implement QVH PTIs. The predominant method entails the reduction from *C*_6_ or *C*_3v_ to *C*_3_ symmetry of the hexagonal unit cell. An energy detuning between the two sublattices within a honeycomb geometry can enable the QVH effect [186–189]. Breaking P symmetry has also manifested the QVH effect in kagome [190–193] and square lattices [194,195], as well as in amorphous lattices lacking long-range periodicity [196].

## Applications of On-Chip THz PTIs

We now focus on recent advancements in THz on-chip PTIs and their applications. While this topic has been previously reviewed [32,96], the rapid progress over the past two years has resulted in many groundbreaking innovations, warranting a renewed focus.

### High-speed communications

In the upcoming 6G and 7G eras, the rapid advancement of THz communication is expected to place even higher demands on THz photonic integrated circuits and high-speed on-chip optical interconnects. These technologies require efficient, low-loss, highly integrated, and robust THz waveguides supporting stable signal transmission. However, traditional waveguide technologies face several challenges, including signal attenuation, bending loss, and extreme sensitivity to minor manufacturing defects. In contrast, PTI waveguides have distinct advantages in single-mode, low-loss, anti-scattering transmission. They are highly stable and reliable in the complex environments typical of on-chip communication systems. Furthermore, PTIs exhibit linear dispersion properties, ensuring excellent performance in broadband communications and reducing the risk of signal distortion.

**Fig. 6.**
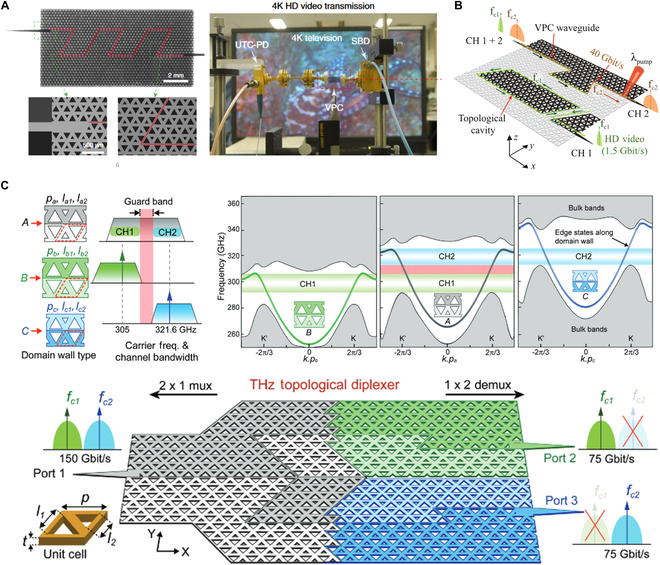
THz on-chip communications. (A) Schematic structure of all-silicon VPC waveguide with a twisted zigzag domain wall marked by red line (left). Real-time transmission of uncompressed 4K high-definition video through VPC (right) [16]. (B) VPC topological demultiplexer [197]. Data signals with carrier frequencies $f_{c1}$ and $f_{c2}$ are demultiplexer to channels 1 and 2, respectively. (C) Design principles of topological diplexer. Three types of VPCs with their supported kink state frequencies (top left). Projected band diagram of zigzag domain wall of three kinds of VPCs (top right). Schematic of diplexer for multiplexing/demultiplexing (bottom) [199].

Yang et al. [16] demonstrated on-chip THz transmission using a topological valley photonic crystals (VPCs) for the first time. This VPC chip was fabricated on high-resistivity silicon due to its extremely low absorption loss. The design involves etching equilateral triangular holes, arranged in a graphene-like lattice, into the silicon substrate (Fig. 6A, left panel). Within the frequency range from 0.32 to 0.35 THz, the transmission coefficient for VPC with a twisted or straight domain wall is close to unity. The estimated loss is smaller than 0.1 dB per bend. As a proof of concept, the researchers demonstrated THz communication with data transmission rates up to 11 Gbit/s and uncompressed 4K video transmission (Fig. 6A, right panel). Subsequently, utilizing orthogonal amplitude modulation at a carrier frequency of around 0.33 THz, valley PTIs have demonstrated the capability to achieve data transmission rates exceeding 100 Gbit/s [197–199]. The growing demand for higher data rates has made exploring frequency bands beyond the conventional range necessary. THz topological transport has been demonstrated at frequencies as high as the 600-GHz band [200].

Frequency-division multiplexing (FDM) facilitates the concurrent transmission of multiple signals by allocating each to a distinct frequency band, optimizing communication capacity, and improving spectrum utilization efficiency. Precisely designing topological characteristics makes achieving highly isolated, multi-channel FDM systems possible, driving THz on-chip communications toward higher speeds and greater integration. In a THz VPC demultiplexer (Fig. 6B) [197], the signal at the carrier frequency $f_{c1}$ is critically coupled into the topological resonant cavity (outlined by the green parallelogram) and output through CH1. In contrast, the nonresonant signal at the carrier frequency $f_{c2}$ is routed through CH2. Perfect isolation can be achieved between the resonant and nonresonant signals. Moreover, a THz topological duplexer chip for multiplexing and demultiplexing has been developed [199]. The lattices' geometric configuration decides the bandgap's frequency range (top left panel of Fig. 6C). A-type domain wall consists of VPC with a broad bandgap. B-type and C-type domain walls consist of VPC unit cells with nonoverlapping bandgap ranges, thereby preventing signal crosstalk. The bandgap ranges of the B-type and C-type domain walls are contained within the bandgap range of the A-type domain wall. The B-type domain wall transmits data signals through CH1, while the C-type domain wall transmits signals through CH2 (Fig. 6C, top right panel). The topological duplexer chip comprises a heterogeneous structure of VPC unit cells with distinct bandgap regions (Fig. 6C, bottom panel). The green and blue regions contain the B-type and C-type domain walls, supporting signal transmission for CH1 and CH2, respectively, while the gray region supports both signals. The photonic duplexer chip supports two independent, well-isolated communication channels, each with a 12.5-GHz bandwidth and nearly flat group delay. The average channel isolation for CH1 and CH2 in the lower and higher frequency bands is 19 and 17.5 dB, respectively. A high-order modulation format achieves a maximum data rate of 75 Gbit/s per channel, resulting in an aggregate data rate of 150 Gbit/s.

### Routers and power splitters

PTIs expedite the development of on-chip functional components, such as routers [190,199,201–205], power splitters [206–208], and directional couplers [209], which are challenging to realize using conventional waveguides. These devices are essential for dynamically managing energy flow in photonic integrated circuits, improving signal interconnection, processing, and communication efficiency and flexibility.

**Fig. 7.**
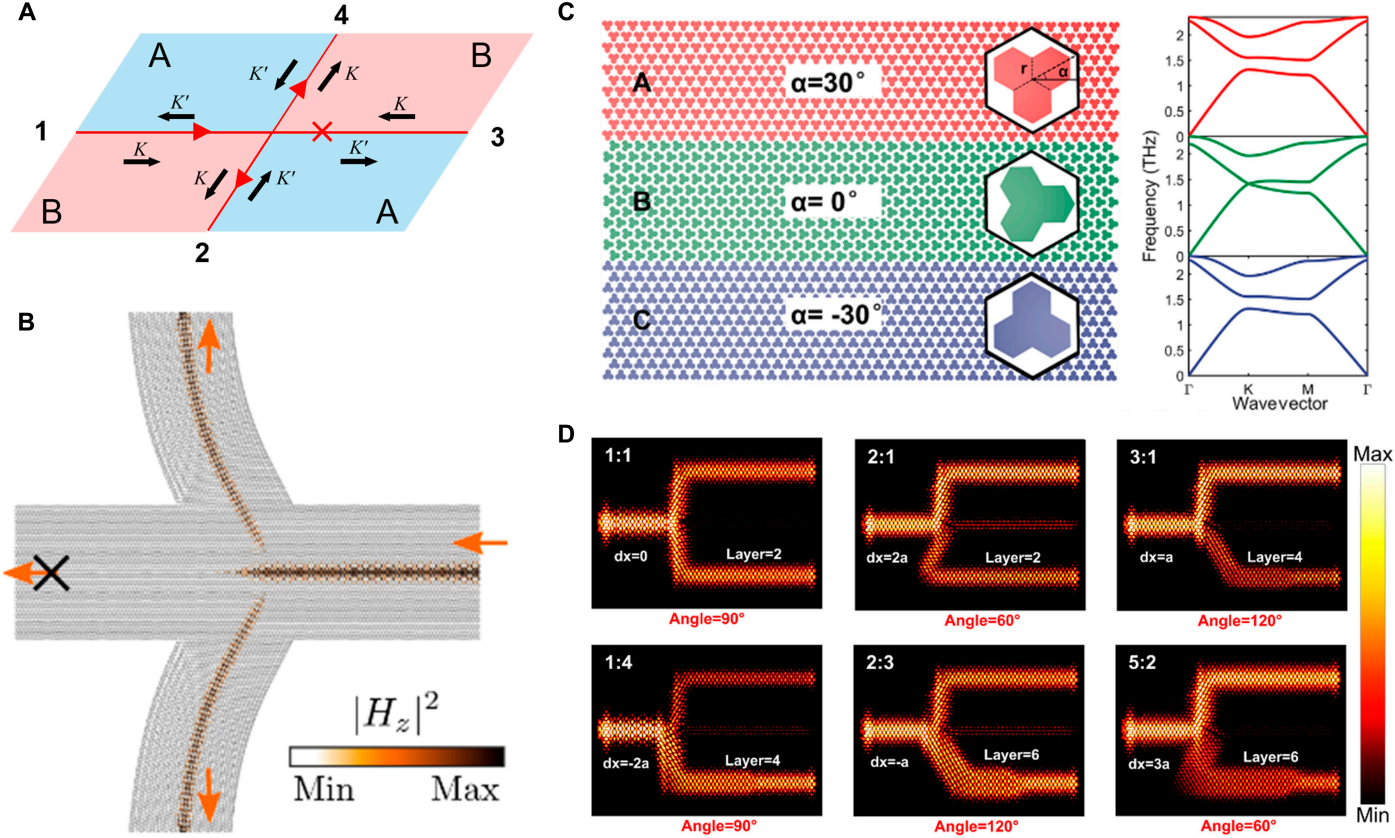
THz on-chip topological routers and power splitters. (A) Principle of valley topological router. Right-propagating kink state at port 1 is locked to *K* valley and only transports to ports 2 and 4. (B) Amorphous topological bend waveguide routes energy into 2 perpendicular directions [211]. (C) Heterogeneous large-area waveguide, where a trivial VPC is sandwiched between 2 VPCs with opposite valley Chern numbers (left) [207]. Dispersion bands of 3 lattices (right). (D) Topological power splitter with arbitrary power splitting ratios [207].

We describe a universal method for routing signals using valley PTIs, where regions A and B, distinguished by opposite valley Chern numbers, are arranged as illustrated in Fig. 7A. A kink state excited at port 1 propagates rightward along the domain wall. The valley-locked nature of this state ensures unidirectional propagation toward ports 2 and 4, while the state propagating toward port 3 is prohibited because it is locked to the *K^'^* valley. By configuring only two PTIs with opposite valley Chern numbers, this design allows for flexible, on-demand routing or splitting of energy flow [209,210].

Hybrid topological phases provide a new scheme for mode routing. Xing et al. [210] designed a twisted Kekulé lattice with bands corresponding to both QSH and QVH phases. The edge states arising from distinct topologies exhibit different behaviors at the terminations of the channels: the helical edge state radiates into free space, while the kink state propagates as on-chip edge modes. Beyond the straight waveguide router, various shapes and curved topological waveguides can be implemented. Like optical fibers, these waveguides enable the flexible interconnection of components that can be distributed arbitrarily across the chip, a feature unattainable with undeformed VPC. Banerjee et al. [211] demonstrated efficient THz transmission through topological waveguides with varying bending angles, utilizing an amorphous lattice structure. This design can route THz waves in two perpendicular directions, as shown in Fig. 7B. Remarkably, despite the amorphous nature of the lattice and the absence of long-range periodicity, the topological protection of the waveguide was maintained through short-range order, ensuring robust, low-loss signal propagation. Controlling power distribution across different channels is a critical area of study. Power splitter with arbitrary splitting ratios can be implemented using topological heterostructures [207], where a VPC with a closed Dirac cone is sandwiched between two VPCs possessing opposite valley Chern numbers (Fig. 7C). The mode width, rotation angle, and lateral displacement of the trivial VPC in the lower channel control power splitting ratios of two channels flexibly (Fig. 7D). This topological waveguides, with adjustable mode width [212–215], can support high-power-density energy transmission and are easily integrated with other on-chip THz components.

### Beam forming/steering

In wireless front-end systems, antennas are core signal transmission and reception components, especially in the THz band. The increased free-space path loss at higher frequencies, combined with the limited output power of current THz sources, underscores the need for high-performance THz antennas. To address these challenges, antennas should have high directivity and gain to offset the high free-space path loss, wide bandwidth to support large channel capacity and meet the demands of high data-rate transmission, and low insertion loss to minimize momentum mismatch with free space.

The primary challenge for on-chip integrated waveguide antennas is the momentum mismatch between waveguide modes and free-space plane waves [216]. This mismatch stems from the refractive index difference between the photonic structure and free space, leading to distinct propagation constants. Traditionally, minimizing reflection at the radiation end-face requires the antenna's input impedance to match the waveguide's characteristic impedance closely. However, it is not necessary to rely solely on impedance matching. By leveraging topological edge states' unidirectional and reflectionless properties, impedance matching can be bypassed, leading to effective output coupling [217]. Moreover, the frequency range of the topological bandgap determines the antenna's operational bandwidth. PTIs with sufficiently wide bandgaps can meet the broadband requirements.

**Fig. 8.**
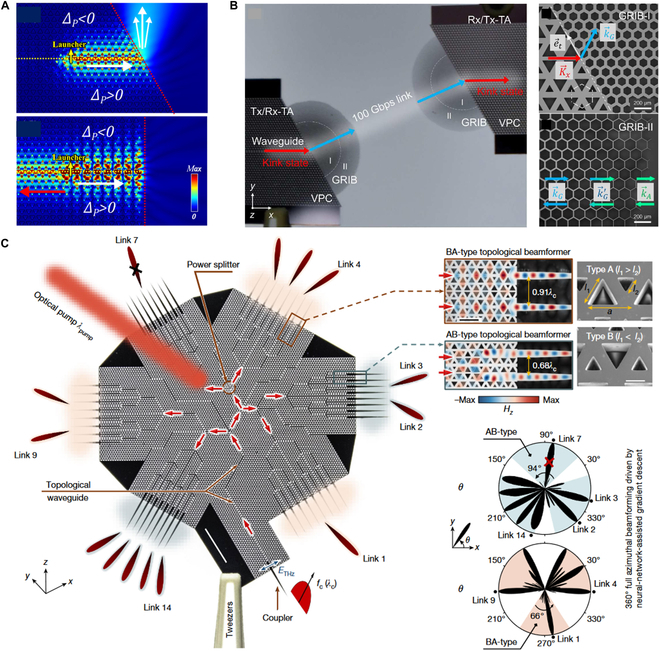
THz topological integrated antennas. (A) Outcoupling of topological kink state with zigzag (top) and armchair (bottom) terminations [185]. (B) Valley-conserved topological integrated antenna used for THz wireless communication link [220]. The transmitter and receiver are composed of VPC waveguides and semi-circular GRIBs (left). Kink state couples into the mode in GRIB-I along with momentum matching and adiabatically transforms in GRIB-II and eventually matches the air momentum (right). (C) THz 360° topological beamformer with 6 branches transmitting signal toward different directions (left) [222]. AB- and BA-type beamformers (top right). Beamformers transmit signals with desired arbitrary azimuthal angles within a range of 94° for AB-type and 66° for BA-type (bottom right).

Kink states can be perfectly coupled into free space upon the valley momentum conservation mechanism. For example, in a VPC waveguide, a valley kink state can radiate into free space through the zigzag termination without back-reflection [190,218]. Although the vacuum does not possess the corresponding valley degrees of freedom, the zigzag termination can suppress inter-valley scattering (Fig. 8A, top panel) [185,219]. This is because the symmetry of the zigzag termination causes the field distributions of edge states from different valleys to be orthogonal. In contrast, the armchair termination lacks this symmetry and causes inter-valley scattering (Fig. 8A, bottom panel). The direction of energy emission into free space depends on the valley to which the kink state is locked because different valleys have opposite transverse momentum.

The direct outcoupling of a kink state from the waveguide into free space exhibits a large divergence angle, which fails to meet high directivity and gain requirements. Jia et al. [220] utilized valley momentum conservation to achieve efficient THz wave conversion between a VPC chip and free space. As shown in Fig. 8B (left panel), the transmitter and receiver in this communication link are both high-gain topological antennas composed of VPC waveguides and semi-circular graded refractive index buffers (GRIBs). The GRIB, a triangular lattice photonic crystal with hexagonal air holes, mitigates the momentum mismatch caused by the high refractive index contrast between silicon and air [221]. At the VPC-GRIB interface, the kink state with momentum ${\vec{K}}_{\;x}$ couples into the GRIB mode with momentum ${\vec{k}}_{\;G}$. In the GRIB-II region, the hole sizes gradually vary along the radiation direction to create an effective refractive index gradient (Fig. 8B, right panel). Upon this, momentum adiabatically transitions from ${\vec{k}}_{\;G}$ to the momentum ${\vec{k}}_{\;A}$ in the air. This antenna achieves a maximum gain of 12.2 dB and a half-power beamwidth of 25° and maintains a stable gain of 12.2 dB over a wide bandwidth range of 30 GHz, ranging from 0.31 to 0.34 THz. As a demonstration of its practical use, an inter-chip wireless THz communication link was realized, with a data transmission rate of up to 100 Gbps.

Furthermore, during signal reception, VPC antennas exhibit directional selectivity akin to dolphin biosonar. They can only capture signals from specific directions without interference from waves originating elsewhere [218]. When the wavevector of an incident wave in free space satisfies the phase-matching condition for a particular valley, the corresponding projected edge state is excited. This directional antenna/receiver has potential applications in encryption communication and anti-jamming technology.

To expand the spatial coverage of signal transmission and improve spectral efficiency, beam steering and beamforming are crucial for the directional transmission of THz signals to spatially separated users. Recently, Wang et al. [222] introduced an on-chip multi-link THz VPC beamformer capable of directing on-chip signals into free space with multiple beams at arbitrary azimuth angles. This beamformer employs VPCs to split the THz signals into six main branches, each separated by 60° in azimuth. Within each branch, the signals are further divided using three-stage power dividers. The neural network-assisted inverse design optimizes the lengths of tapered structures to control the spatial phase distribution of the radiated waves, enabling the generation of desired beam patterns. Kink states within the VPC chip ensure robust wave propagation while suppressing crosstalk between adjacent channels. Two topological beamformers, AB-type and BA-type (Fig. 8C, top right panel), offer azimuthal coverage ranges of 94° and 66°, respectively. They provide full 360° omnidirectional coverage (Fig. 8C, bottom right panel). Chip-to-chip wireless communication experiments over a distance of 300 mm with a data rate of 72 Gbit/s have been demonstrated using a pair of such topological beamformers. This work presents a promising solution for large-scale beamforming and multi-input multi-output systems in terabit-per-second wireless communications, paving the way for more efficient 6G and future XG networks​​​​.

### Quantum cascade lasers

QCLs are compact semiconductor lasers that operate in the mid-infrared and THz frequency ranges [223–227]. THz QCLs are one of the most important and efficient THz sources. However, these photonic cavities are susceptible to defects, disorder, and fabrication imperfections, which limits their performance and applicability. Recent studies have demonstrated that various topological edge states can be leveraged to construct topologically protected laser cavities [228–233].

**Fig. 9.**
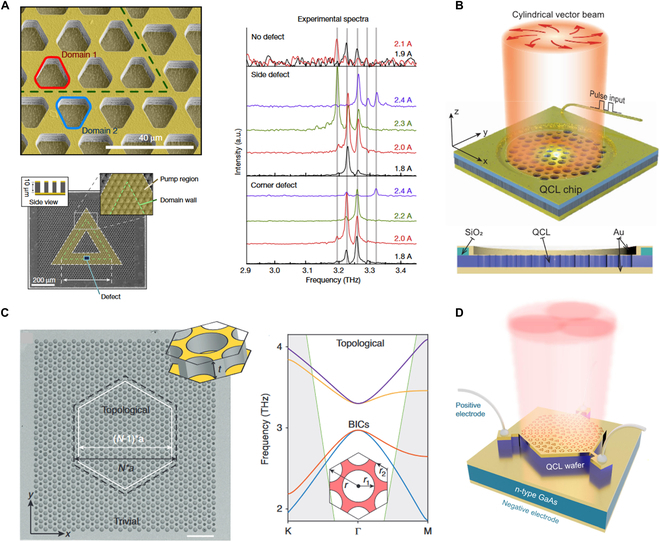
Electrically pumped THz topological QCLs. (A) THz topological QCL cavity is composed of a triangular loop (left) [234]. The yellow shaded area is the pumped area, the green dashed line is the domain wall, and the black rectangle is the defect to outcouple lasing modes vertically. Emission spectra have no outcoupling, side, and corner defects (right). (B) THz QCL based on an MZM emits a cylindrical vector beam [237]. (C) Laser cavity of topological bulk BIC QCL, with pump area inside the white hexagon (left) [238]. Band structure of topological nontrivial lattice after band inversion, where the degenerate quadrupolar modes (orange and blue lines) appear as BICs (right). (D) High-power SMDC QCL without graphically etched active region [240].

The first electrically pumped THz topological QCL was experimentally demonstrated by Zeng et al. [234]. This work inscribed a triangular lattice of hexagonal holes onto a THz QCL wafer, forming domain walls supporting valley kink states along a triangular ring (Fig. 9A, left panel). The laser cavity comprises this triangular loop, where the kink states circulate robustly. The yellow shading indicates the electrically pumped areas, while the unshaded areas are not subjected to injection. A defect in the form of a black rectangle etched through the active medium enhances the vertical outcoupling efficiency of the in-plane lasing modes. Remarkably, even when perturbations occur along the sides or corners of the triangular cavity, uniformly spaced laser emission peaks are maintained (Fig. 9A, right panel). This robustness is attributed to the ability of the topologically protected kink states to propagate without localization.

Topological cavities have also been demonstrated to enable lasers with nontrivial emission modes [235,236]. Han et al. [237] demonstrated a photonic analog of Majorana zero mode (MZM) on THz electrically pumped QCL. As shown in Fig. 9B, the topological cavity is implemented by an air-hole hexagonal lattice drilled through the top contact metal layer and the active medium of the QCL wafer. A vortex-like Kekulé modulation is applied to the lattice to induce inter-valley coupling and generate a 2π phase with a winding number of +1. The QCL wafer provides a gain in the range of 2.9 to 3.8 THz, which overlaps with the designed photonic bandgap. Under electrical pumping, the far-field beam profile exhibits a cylindrical vector beam characterized by a doughnut-shaped intensity distribution. A topological bulk BIC THz QCL has been developed by combining topological phases with BIC [238] (Fig. 9C, left panel). This design offers several advantages, such as miniaturization—the laser size is approximately three wavelengths—and efficient single-mode emission with a side-mode suppression ratio of 20 dB. In the nontrivial topological lattice, band-inversion quadrupole modes at the center of the Brillouin zone manifest as symmetry-protected BICs (Fig. 9C, right panel). The contrast between trivial and nontrivial topological phases leads to lateral confinement of lasing modes within the cavity. Simultaneously, the bulk BIC engages in out-of-plane tight confinement. The gain competition ensures the laser's single-mode output characteristics. Moreover, the topological charge of BIC causes the laser to exhibit a cylindrical vector beam in the far-field emission [239].

In earlier designs, patterns etched into the active region of THz QCLs often resulted in reduced output power. To address this limitation, Liu et al. [240] proposed a surface metal Dirac-vortex cavity (SMDC) design, wherein the topological cavity is positioned on the surface metal layer. This configuration preserves the integrity of the active region, providing sufficient gain for high-power output. The unit cell of the topological cavity consists of a hexagonal supercell honeycomb lattice formed by 6 adjacent sites (Fig. 9D). The strong coupling between the SMDC and the active region generates a robust 2D topological defect laser mode. Furthermore, the introduction of Kekulé phase modulation enables vortex-polarized far-field emission. This SMDC design prevents damage to the active region and achieves a single-mode surface-emitting peak power of 150 mW. These electrically pumped topological lasers, characterized by high radiation efficiency, offer potential for practical applications in 6G wireless communication [241]. Table 3 quantifies the performance of various THz QCLs. It can be seen that THz QCLs based on topologically protected photonic crystal cavities exhibit advantages over conventional photonic crystal and other types of microcavities in terms of tunability of far-field radiation patterns, robustness, and output power.

**Table 3.** Performance comparison of several types of THz QCLs

Laser cavity	Year	Emission frequency (THz)	Operating temperature (K)	Output power	Main features
Photonic crystal [292]	2009	~2.7	60	/	Single-mode
Inductor–capacitor resonant circuit [293]	2010	1.5	10	16 nW	Ultra-compact footprint *V* ≈ *λ*^3^/100, low threshold ~1.1 mA
Microdisks [294]	2017	3.5	10	220 μW	Ultra-compact footprint *V* ≈ *λ*^3^/100, low threshold ~6 mA
Topological [234]	2020	~3.2	9	/	High robustness, compact footprint
Topological [237]	2023	~3.4	8.5	9.04 mW	Single-mode, vector beam
Topological [238]	2023	2.93	8.5	/	Single-mode, compact footprint ~3*λ*, vector beam, high side-mode suppression ratio ~20 dB
Topological [240]	2024	~3.3	13	150 mW	High power, single-mode, vector beam

### Optical delay lines

Data buffering is critical in optical communication and computational networks [242]. Unlike electrical signals, optical signals cannot be directly stored in a conventional medium, necessitating all-optical data buffering solutions, typically achieved through various tunable optical delay lines. Integrated optical delay lines can be implemented using single or cascaded microring resonators [243], waveguide gratings [244], photonic crystals [245], and looped circuits [246]. The key performance metric for an optical delay line is the optical delay time $\Delta t=n_{g}L∕c$, where *L* presents the optical path length, *n_g_* is the group index, and *c* is the speed of light in the vacuum. Conventional optical delay lines face limitations in achieving long delays and broadband operation within a compact footprint.

A breakthrough in this field is the development of topological delay lines, which leverage topologically protected edge states to address these challenges. These edge states allow light to propagate with minimal loss, even through multiple sharp bends. This enables extended optical path lengths while maintaining device compactness [185,198]. The linear dispersion of these edge states supports higher signal fidelity and larger bandwidths. Additionally, the flexible design of topological delay lines permits their integration into photonic circuits.

**Fig. 10.**
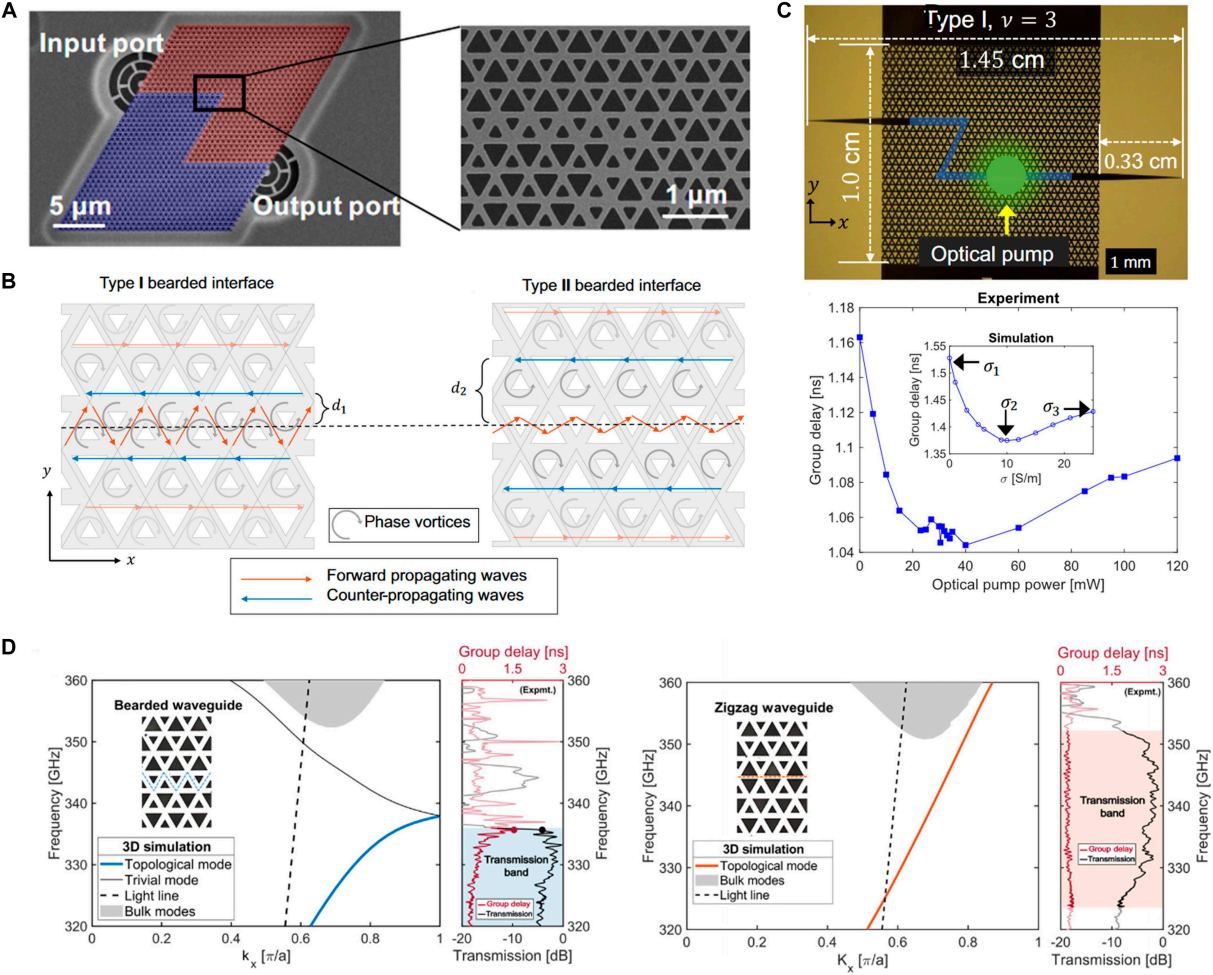
Topological delay lines. (A) Bearded interface VPC waveguide exhibits a slow-light effect [253]. (B) Magnetic phase vortex (gray ring)-induced counter-propagating waves (blue arrow) interact with forward propagating waves (orange arrow), giving rise to a slow-light effect [255]. (C) Tunable slow light of bearded interface waveguide [255]. Group delay is tuned by optically exciting carriers at the all-silicon VPC waveguide interface. (D) Band diagram, group delay, and transmission coefficients of bearded (left) and zigzag (right) interface VPC waveguides [257].

The first topological delay line was demonstrated using a lattice of coupled ring resonators in a QSH system [247], which shows resilience to defects and maintains performance despite intentional fabrication imperfections. Other topological systems can also be employed to design broadband delay lines based on the QVH effect. A common strategy to achieve longer delay times is increasing the path length of edge states within a small footprint. Another approach for delay line design depends on the slow-light effect in topological edge states: increasing the group index *n_g_* by reducing the group velocity [248–251]. Recently, the slow-light effect has been discovered in bearded interface VPC waveguide [252,253], as illustrated in Fig. 10A. Owing to phase vortices in the VPC, the optical path length of kink states along the propagation direction exceeds that of traditional strip waveguides [254]. The zigzag interface, characterized by mirror symmetry, supports only forward-propagating kink states. In contrast, the bearded interface, featuring glide symmetry, allows the coexistence of both forward and backward propagating waves (Fig. 10B) [255]. These backward-propagating waves arise from the interaction between magnetic vortices and the glide-symmetric bearded interface. Before the forward-propagating kink states progress, the backward-propagating waves temporarily trap the kink states within each magnetic vortex, thus leading to a reduction in group velocity. By engineering the interface and bandgap of the VPC waveguide, the coupling strength between forward and backward-propagating waves can be precisely modified [256]. Furthermore, active control of topological slow-light effects has been demonstrated by optically exciting carriers at the all-silicon VPC waveguide interface (Fig. 10C) [255].

The trade-off between high group delay and reduced robustness is a fundamental principle regarding the stability of topological modes in photonic crystals [257]. In bearded interface waveguides, a decrease in group velocity leads to a nonlinear increase in group delay, with the maximum slow-light effect occurring near the edge of the topological bandgap (Fig. 10D, left panel). Conversely, the linear dispersion of zigzag interface waveguides results in lower and relatively uniform group delay values (Fig. 10D, right panel). In bearded interface waveguides, the group delay is closely associated with non-Hermitian loss modulation, where high group delay values correspond to increased sensitivity to non-Hermitian loss. This phenomenon is attributed to the slowness-induced enhanced interaction of the waveguiding modes with the photoexcited carriers. VPC waveguides with zigzag interfaces are well suited for interconnects due to their robustness and uniform group delay. In contrast, bearded interfaces supporting slow-light states are ideal for exploring topological light–matter interactions and THz on-chip modulators.

### Reconfigurable THz PTIs and potential applications

As photonic applications become increasingly complex, static topological structures are often insufficient to meet the evolving requirements of optical communication and information processing. To address this, reconfigurable topological photonics has emerged, showing considerable potential in areas such as optical communication [97], quantum computing [258], and intelligent recognition [259]. This section explores recent advancements in reconfigurable PTIs and prospects for their potential applications in the THz regime. Reconfigurability is primarily achieved through electrical, optical, mechanical, and thermal control mechanisms.

**Fig. 11.**
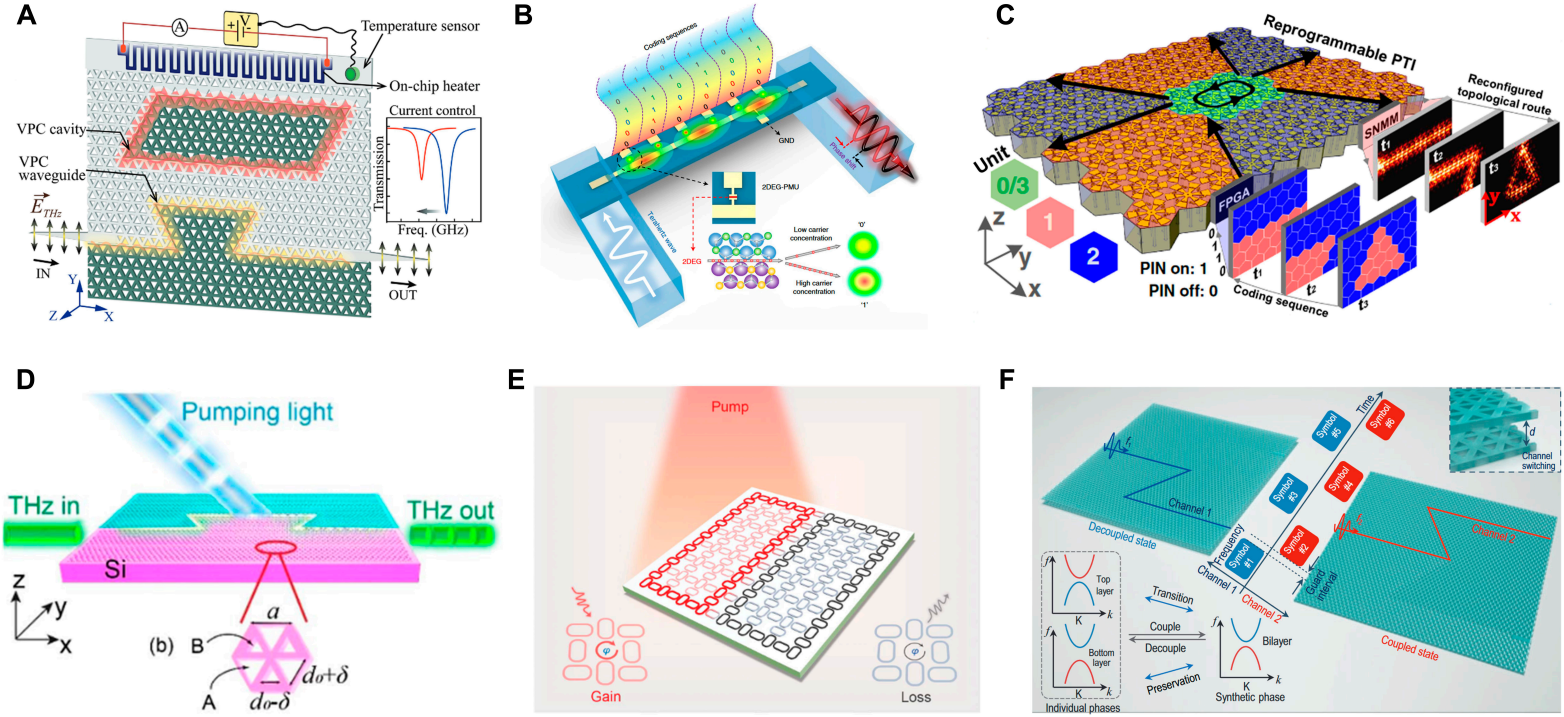
Reconfigurable PTIs. (A) THz topological cavity with electrically tunable frequency shift [267]. (B) Digitally programmable THz on-chip phase controller [268]. (C) Electrically reprogrammable enabled ultrafast topological propagation route control [269]. (D) Optically controllable THz topological transmission switching through free silicon carriers excited by pumping light [272]. (E) Optical pump-induced local non-Hermitian control of light propagation in topological PTIs [274]. (F) Mechanical modulation of THz on-chip PTIs in time-frequency interleaved complex domain [275].

In electrically reconfigurable PTIs, materials such as liquid crystals [260–263], graphene [264,265], and barium titanate [266] dynamically respond to external stimuli. These materials primarily modulate the refractive index (or Fermi energy, in the case of graphene), allowing precise tuning of the frequency and bandwidth of the topological edge states. Low-loss nematic liquid crystals are critical for practical THz applications to ensure efficient transmission. Moreover, integrating these materials with all-silicon platforms presents great potential for further technological advancement. Gupta et al. [267] recently introduced an electrically tunable on-chip THz topological notch filter. This device tunes the frequency of topological cavity mode via current control, achieving a 1-GHz frequency shift with MHz-level resolution (Fig. 11A). The integration of 2D electron gas (2DEG) offers a promising approach to THz on-chip components. Zeng et al. [268] demonstrated a highly precise, digitally programmable THz on-chip phase shifter by controlling electron transport in the 2DEG with an external voltage, thereby modulating the phase of guided waves (Fig. 11B). Although this device does not incorporate topological features, it provides valuable insights for the further development of topological THz phase modulators.

Other electrically programmable PTIs rely on the integration of positive intrinsic negative (PIN) diodes. You et al. [269] introduced an ultrafast reconfigurable plasmonic PTI (Fig. 11C), in which a field-programmable gate array network controls the switching of PIN diodes at nanosecond speed. The interfaces formed by differently encoded units support topological edge states. The transmission paths can be reconfigured by altering the electrical coding. Although the integration of lumped elements with THz topological circuits has not yet been realized, this remains a promising approach for developing programmable THz PTIs.

Optical excitation offers distinct advantages for modulating PTIs, such as noncontact operation and high-speed response. In all-silicon PTIs, optical pump-induced free carriers can introduce non-Hermitian loss [197,222,255,270–272]. By focusing pumping light on the interface, localized edge states can be generated and effectively shut down communication channels [272] (Fig. 11D). If the pump pulses are short enough, high-speed topological optical switches with switching times in the nanosecond range can be achieved [270]. At higher pump fluences, the thermal energy from optical conversion alters the refractive index of silicon, thus leading to frequency tuning of THz topological resonators [271]. Optical carriers can affect the group velocity of kink states within VPC waveguides [255,257]

Beyond silicon, transparent conductive oxides (TCOs) and indium gallium arsenide phosphide (InGaAsP) have also demonstrated the ability to modulate topological properties. Lightly doped TCOs exhibit dielectric responses in the near-infrared band, modulating their refractive index via electrical or optical excitation [273]. InGaAsP quantum well semiconductors, when optically pumped, enter a gain state, while in the absence of pumping, they revert to a high-loss state. Controlling the gain–loss difference across non-Hermitian EP can result in unidirectional topological edge states at the boundary of the gain region. The topological boundaries are reconfigurable and can be flexibly controlled by tailoring the pump light pattern (Fig. 11E) [274].

Mechanically reconfigurable THz PTIs can be realized by tuning the interlayer coupling distance between bilayer VPCs (Fig. 11F) [275]. This allows for controllable switching between coupled and decoupled states, enabling reconfigurable channel switching. Specifically, a large interlayer distance decouples two VPCs, and the system transmits signals with frequency $f_{1}$ through channel 1. The two VPC layers couple as the distance decreases, and signals with frequency $f_{2}$ are transmitted through channel 2. The mechanical tuning process offers a time-domain guide interval, achieving time-frequency interleaved THz on-chip modulation.

In addition, temperature-sensitive materials, such as phase-change materials such as vanadium dioxide [276,277], germanium–antimony–tellurium (GST) [278,279], antimony sulfide and antimony selenide [280], ferroelectric materials [281], and silicon [282], have also been utilized in reconfigurable PTIs. Thermal-induced phase transitions change the refractive index or conductivity of these materials. For instance, GST-based metamaterial can rapidly switch its topological edge states within hundreds of nanoseconds under thermal modulation [279]. The transition in GST nanopatterns attached to the lattices results in band inversion in QSH PTIs and directly changes the spin Chern number [278]. VO_2_ has already been used to modulate topological edge states' on/off switching [276,277].

## Conclusion

This review presents an overview of quantum-inspired THz metamaterials, focusing on their development and applications for spatial and on-chip THz waves. First, we discuss the physical principles and phenomena associated with quantum-inspired metamaterials designed for spatial THz waves, covering fundamental concepts of EIT, BICs, Fano resonances, nonhermiticity, and EPs. We further show that those concepts can be employed in THz waves for numerous functional devices for spatial THz waves—including efficient modulators, highly sensitive sensors, nonlinear THz sources, and perfect absorbers. We also review on-chip quantum-inspired topological metamaterials and elaborate on band topologies underlying QH, QSH, and QVH PTIs. We further highlight applications of on-chip THz PTIs in THz communications and several integrated functional components, such as routers, power splitters, antennas, QCLs, and delay lines. Finally, we briefly review the progress made in reconfigurable THz PTIs and their prospective applications.
